# Gene expression profiles during short-term heat stress; branching *vs.* massive Scleractinian corals of the Red Sea

**DOI:** 10.7717/peerj.1814

**Published:** 2016-03-28

**Authors:** Keren Maor-Landaw, Oren Levy

**Affiliations:** The Mina and Everard Goodman Faculty of Life Sciences, Bar-Ilan University, Ramat Gan, Israel

**Keywords:** Coral, Gene expression, Heat stress, Branching coral, Massive coral, Coral morphology, Hsps, Oxidative stress, ER stress

## Abstract

It is well-established that there is a hierarchy of susceptibilities amongst coral genera during heat-stress. However, molecular mechanisms governing these differences are still poorly understood. Here we explored if specific corals possessing different morphologies and different susceptibilities to heat stress may manifest varied gene expression patterns. We examined expression patterns of seven genes in the branching corals *Stylophora pistillata* and *Acropora eurystoma* and additionally in the massive robust coral, *Porites* sp. The tested genes are representatives of key cellular processes occurring during heat-stress in Cnidaria: oxidative stress, ER stress, energy metabolism, DNA repair and apoptosis. Varied response to the heat-stress, in terms of visual coral paling, algal maximum quantum yield and host gene expression was evident in the different growth forms. The two branching corals exhibited similar overall responses that differed from that of the massive coral. *A. eurystoma* that is considered as a susceptible species did not bleach in our experiment, but tissue sloughing was evident at 34 °C. Interestingly, in this species redox regulation genes were up-regulated at the very onset of the thermal challenge. In *S. pistillata*, bleaching was evident at 34 °C and most of the stress markers were already up-regulated at 32 °C, either remaining highly expressed or decreasing when temperatures reached 34 °C. The massive *Porites* species displayed severe bleaching at 32 °C but stress marker genes were only significantly elevated at 34 °C. We postulate that by expelling the algal symbionts from *Porites* tissues, oxidation damages are reduced and stress genes are activated only at a progressed stage. The differential gene expression responses exhibited here can be correlated with the literature well-documented hierarchy of susceptibilities amongst coral morphologies and genera in Eilat’s coral reef.

## Introduction

Over the past several decades, corals throughout the world have been affected by sea surface temperature (SST) anomalies associated with global warming (Hoegh-Guldberg et al., 2007). The core morphological and physiological response to thermal stress is termed coral bleaching (Coles & Brown, 2003), and is associated with the mass expulsion (Brown, 1997a), digestion (Downs et al., 2009) and/or suppression of the pigment synthesis (Weis, 2008) of the unicellular photosynthetic dinoflagellate symbionts. Frequent episodes of high SSTs induce severe coral bleaching events, which typically lead to coral death (Brown, 1997a; Hoegh-Guldberg, 2010). Even though the forecast of continuing coral bleaching and mortality is grim, coral susceptibility to heat stress is highly variable and there are indications that some corals can thrive even at temperature extremes (Coles & Brown, 2003; Weis, 2010).

Many mechanisms have been suggested to explain differential bleaching susceptibilities; these hypotheses can be grouped into three categories. The first explanation involves the possibility that the genotype of the algal endosymbiont (*Symbiodinium*) affects the holobiont’s thermal tolerance via effects on photosynthetic dysfunction (Rowan et al., 1997; Baker, 2001; Berkelmans & Van Oppen, 2006; Hawkins et al., 2014). Recent work by Hume et al. (2015) supports the genotypic difference hypothesis as they discovered a new *Symbiodinium* species, *Symbiodinium thermophilum*, prevalent in corals in the world’s hottest sea; southern Persian/Arabian Gulf. The symbiotic dinoflagellate may be able to utilize the xanthophyll cycle as a photoprotective mechanism by dissipation of excess excitation energy (Brown et al., 1999). An additional explanation is that it is the dynamic physiological characteristics of the host which are including its photoprotective mechanisms (MAAs) (Shick & Dunlap, 2002; Lesser, 2004), changes in fluorescent pigments (FP) (Salih et al., 2000), regulation of heat-shock proteins (Black, Voellmy & Szmant, 1995; Hayes & King, 1995; Downs et al., 2000; Richier et al., 2006) and antioxidant enzymes, that mitigate oxidation damage (Baird et al., 2009), differential regulation of host apoptosis (Tchernov et al., 2011) and/or generation of nitric oxide (NO) (Hawkins et al., 2014) in reaction to stress. The third hypothesis also includes the importance of the coral’s thermal history aiding in acclimation and increasing its capacity for mitigating cellular stress (Brown et al., 2002; Barshis et al., 2010; Weis, 2010).

There is a hierarchy of susceptibilities amongst coral genera during heat stress (Harriott, 1985; Glynn, 1988; Cook et al., 1990). Branching species, especially acroporids, are generally known to be more susceptible to bleaching when compared to massive corals, such as *Porites* (Jokiel & Coles, 1974; Brown & Suharsono, 1990; Loya et al., 2001). This phenomenon is consistent over wide geographic ranges and was documented in Hawaii (Jokiel & Coles, 1974), Java Sea (Brown & Suharsono, 1990), Japan (Loya et al., 2001) and the Great Barrier Reef (Marshall & Baird, 2000). However, an exception from that pattern was observed in juvenile *Acropora* colonies. This finding was explained by the colony size: *Acropora* colonies <5 cm are often flat prior to branching and forming 3-dimensional structures, hence will survive better than larger colonies (Loya et al., 2001; Van Woesik et al., 2011). Moreover, short-term response will not necessarily apply to the long-term and recovery behavior that is depended on number of variables (Van Woesik et al., 2011).

To date there have been few studies aimed at explaining the discrepancy in bleaching susceptibilities between massive and branching forms in physiological terms. One possible explanation is that massive corals, on average, have thicker tissues than branching species (Loya et al., 2001). These thick tissues may posses more photoprotective abilities through self-shading properties, especially when the polyps are retracted (Hoegh-Guldberg, 1999; Brown, 1997b). Higher densities of fluorescent proteins, known to reduce photoinhibition in corals, and documented in poritids and other less-susceptible taxa, may be an additional explanation (Salih, Hoegh-guldberg & Cox, 1998; Baird et al., 2009). It is also possible that colony morphology influences flow regimes and the differences in boundary layers affect differences in mass transfer at the tissue water interface (Nakamura & Van Woesik, 2001; Loya et al., 2001; DeSalvo et al., 2010b). The light-absorbing properties of the zooxanthellae symbiont were suggested to be effected by colony morphology, shape and size (Enríquez, Méndez & Iglesias-Prieto, 2005; Stambler & Dubinsky, 2005). Furthermore, differences in morphological and physiological features between massive corals and branching species were correlated to more effective acclimatization abilities in these species (Gates & Edmunds, 1999). Coral “losers” i.e., those with branching morphologies (Loya et al., 2001), exhibit high symbiont flexibility (generalist), while massive “winner” corals are often symbiont specific (Putnam et al., 2012). Host flexibility to symbionts under environmental stress, may drive competitive interactions and impair the overall function of the symbiotic interactions resulting in damaged holobiont fitness (Putnam et al., 2012). Furthermore, massive corals resilience can also be explained by a compensating mechanism of increasing heterotrophic feeding and decreasing energy allocated to calcification (Grottoli, Rodrigues & Palardy, 2006; Levas et al., 2013). Recently, Wooldridge (2014) argued that the differences in susceptibilities are due to different strategies of ensuring a continuity of CO_2_ for photosynthesis. Only one attempt was made (DeSalvo et al., 2010b) to compare gene expression profiles (using microarray) of massive and branching corals following similar thermal stress experiment. The authors found that in the massive *Orbicella faveolata* and the branching *Acropora palmata* despite the small gene overlap between the two microarrays (10%), and despite the fact that the percentage of annotated differentially expressed genes was different, there were similar core responses for the two species including an increase of multiple heat shock protein and antioxidant transcripts, a decrease in expression of calcium homeostasis proteins and ribosomal proteins, and changes in the extracellular matrix and in actin cytoskeleton (DeSalvo et al., 2010b). In addition, DeSalvo et al. (2010b) identified expression of markers in *A. palmata* that did not appear in *O. faveolata* including markers for osmotic stress, p53 and NF-*κ*B signaling, sensory perception, the glyoxylate cycle, and nitric oxide signaling.

The gene expression profiles and molecular mechanisms governing the differences in branching vs. massive coral bleaching susceptibilities are still poorly understood. Most of the gene expression studies in Cnidaria that relate to global climate change have been conducted on branching corals (examples: DeSalvo et al., 2008; Rodriguez-Lanetty, Harii & Hoegh-Guldberg, 2009; Pernice et al., 2011), while only about a quarter focused on massive corals such as *Porites* and *Orbicella* (Edge et al., 2005; DeSalvo et al., 2008; Polato et al., 2010; Kenkel et al., 2011; Kenkel, Meyer & Matz, 2013) that are considered to be relatively resilient to heat-stress (Jokiel & Coles, 1974; Brown & Suharsono, 1990; Loya et al., 2001). Indeed corals of the genus *Porites* are one of the most common targets for paleaoclimate studies using cores taken along the coral’s major growth axis. These studies allow the investigation of sea surface temperature, pH, salinity, winds and upwelling, cloud cover, ocean mixing and river discharge histories, can be reconstructed (Grottoli & Eakin, 2007).

In this research we attempt to ascertain if specific corals with different morphologies would have diverse responses to heat stress, in terms of gene expression. We hypothesized that these will have varied gene expression patterns occurring following controlled short-term heat stress. We expect that corals known as relatively sensitive would have a stronger reaction of heat stress markers. So, we tested this in three selected highly abundant coral species of the Gulf of Eilat, the Red Sea (Shaked & Genin, 2015), in which we had also permits to collect, the branching *Stylophora pistillata* and *Acropora eurystoma* and massive robust coral *Porites sp.* These corals grow in relatively shallow waters and are therefore subjected to daily and seasonal water temperature changes (Shaked & Genin, 2015). We explored the expression patterns of seven genes, representatives of key cellular processes occurring during stress in Cnidaria including those in charge of redox regulation, heat shock, energy metabolism, DNA repair, and apoptosis (Maor-Landaw & Levy, in press). These processes (excluding the apoptotic caspase 3) were previously defined as a part of a minimal cellular stress proteome that is highly conserved throughout the metazoan (Kültz, 2005).

## Material and Methods

### Sample collection and experimental design

**Figure 1:**
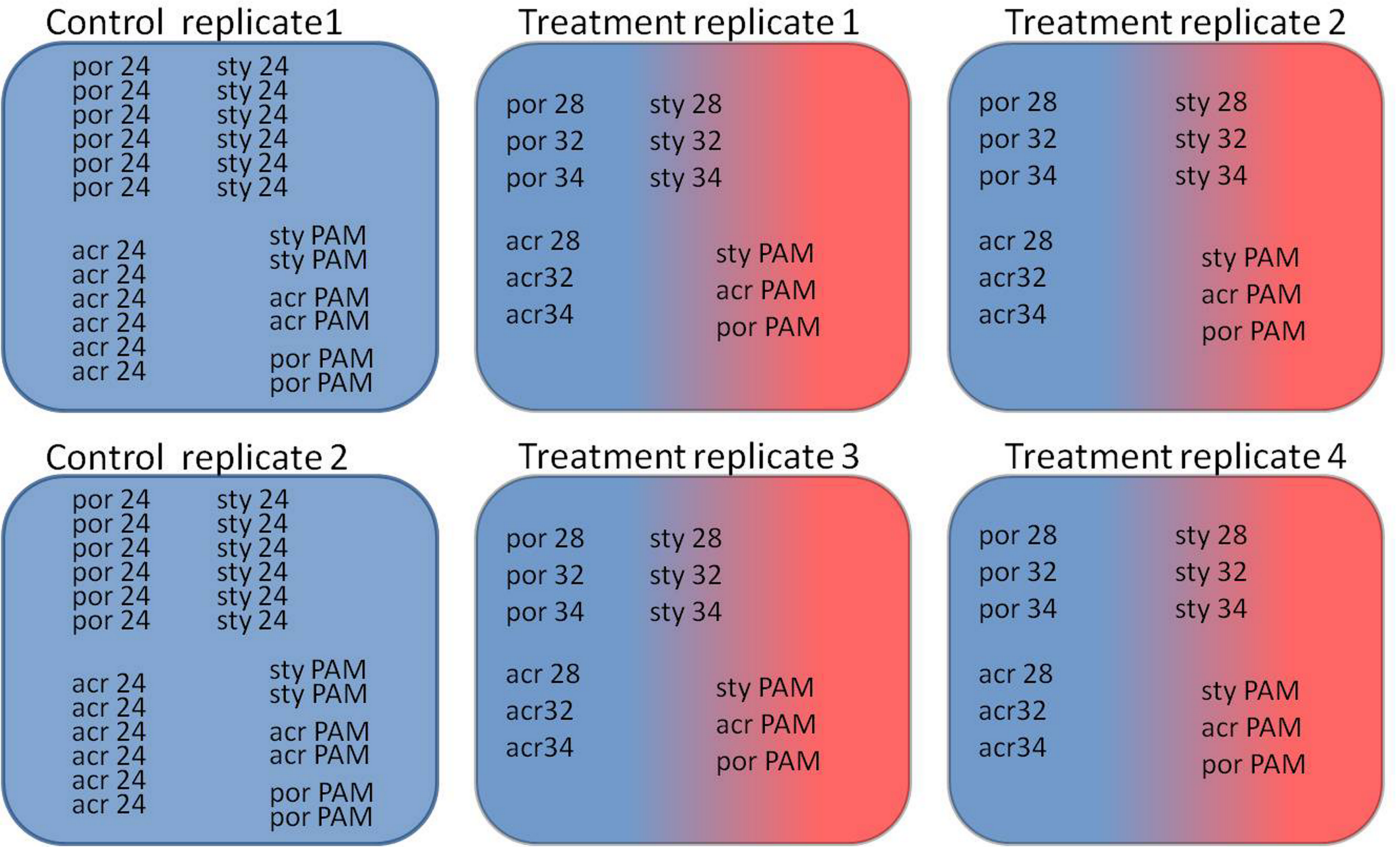
Experimental design of the experiment. Two control aquaria and four experimental aquaria: Coral fragments (por; *Porites sp.*, sty; *S. pistillata*, acr; *A. eurystoma*) were sampled at the time points corresponding to 24 h incubation at the following temperatures (28, 32 and 34; 28 °C, 32 °C and 34 °C) and concurrent PAM measurements of fragments was used for evaluating the maximum quantum yield (PAM; fragments used for evaluating the maximum quantum yield).

During November 2013 and January 2014 single colonies of the branching corals *Stylophora pistillata* and *Acropora eurystoma*, as well as a colony of the massive *Porites sp.* were collected using SCUBA from a depth of 10 m in the Gulf of Eilat (Red Sea) (the Israeli Nature and National Parks Protection Authority approved the collection of corals in this study, permit No. 2013/40159). The upper branches of each the *S. pistillata* and *A. eurystoma* colonies were cut providing 32 fragments each, those were approximately 5 cm in length. A 25 cm-sized *Porites sp.* was fragmented using a core-forming drill into 32 fragments. By fragmenting single colonies, we established duplicate ‘micro-colonies,’ eliminating unwanted sources of genetic and biological variability (Granados-Cifuentes et al., 2013; Hemond, Kaluziak & Vollmer, 2014; Parkinson et al., 2015) derived from colony size, shapes and thermal/light life histories (Tambutté et al., 1995; Brown et al., 2002). In April 2014, following an acclimation period of at least two months at 24 °C in an indoor aquarium at Bar-Ilan University, the fragments were placed randomly in six indoor aquaria (see Fig. 1): two control aquaria (8 fragments from each coral species at each aquarium) were maintained at 24 °C and four experimental aquaria (4 fragments from each coral species at each aquarium) were subjected to a temperature increase of 1 °C per day from 24 °C to 34 °C. The aquaria were maintained with continuous water flow (artificial seawater (Brightwell Aquatics, Pennsylvania, USA)) using a computer-controlled closed circulation system, which compensates for salinity fluctuations and water level changes (constant salinity level of 35‰). Light periodicity was achieved using an Advanced Control Lighting System (ACLS, Sfiligoi, Italy) with HQI (hydrargyrum quartz iodide) light bulbs (400 w, 14,000 Kelvin) configured to simulate a year-long diurnal-dimming light regime (PAR (photosynthetically active radiation) of 150 µmol quanta m^−2^ s^−1^). Four fragments, were sampled at the same time of day from each the control and heat treated aquariums at the time points corresponding to 24 h incubation of: 28 °C (day 5 from the beginning of experiment), 32 °C (day 10) and 34 °C (day 13) (see Fig. 1). The four treatment fragments were sampled from four independent aquaria. In contrast, the four control fragments were sampled from only two aquaria. Our long previous experience with our controlled system suggests that (example Maor-Landaw et al., 2014) there is no difference between the independent aquaria. Therefore, in this experiment the four control fragments sampled from two aquaria were considered as four replicates.

### PAM flourometry

An imaging pulse amplitude modulation (IPAM) fluorometer (Heinz Waltz GmbH, Germany) was used to evaluate the maximum quantum yield of photosystem II in the algal symbionts. The fluorescence of four fragments of each coral species was measured following 30 min of darkness-acclimation following 24 h at each temperature point—28 °C, 32 °C and 34 °. Thus the time points of sampling corresponded to days 0, 5, 10 and 13, at the same time of the day as the sampling for RNA extraction. The maximum quantum yield (*Fv*∕*Fm*) was calculated for each sample by determining the dark-level fluorescence yield (*F*0) and the maximum fluorescence yield (*Fm*) when all PSII reaction centers were photochemically reduced [*Fv*∕*Fm* = (*Fm* − *F*0)∕*Fm*]. The maximum quantum yield helped in monitoring the photosynthetic performance during the experiment, which is an indicator of thermal stress in the symbiont (Fitt et al., 2001; Ainsworth et al., 2008).

### RNA extractions

Extraction protocols changed according to coral species and growth form. Total RNA was extracted from each fragment of *S. pistillata* and *A. eurystoma* using Trizol (Invitrogen Life Technologies, Carlsbad, CA, USA) according to the methods presented in (Levy et al., 2011), and the samples were further purified using a RNA Clean and Concentrator kit (Zymo Research Corp., Irvine, CA, USA). Total RNA was extracted from *Porites* fragments using RNAqueous 4-PCR kit (Ambion), as described by (Kenkel et al., 2011). RNA quantity was assessed using a NanoDrop spectrophotometer (ND-1000). RNA integrity was checked via a Bioanalyzer (Agilent) or alternatively through agarose gel electrophoresis and evaluated based on clear 28S and 18S ribosomal RNA bands.

### Primer design

We examined the expression of six genes: thioredoxin, peroxiredoxin, DNAJ, heat shock protein 70, enolase and Rad51, which were up-regulated following heat-stress in our previous *S. pistillata* study (Maor-Landaw et al., 2014) and also examined the expression of caspase 3. With the exception of *S. pistillata*caspase 3 that was adapted from (Kvitt et al., 2011) the primers for amplifying *S. pistillata* target genes of interest (GOI) were designed based upon *S. pistillata* EST libraries previously constructed in our lab (Karako-Lampert et al., 2014). Degenerate primers were designed for *Porites sp.* and *A. eurystoma* GOI using *Porites astroides* (Kenkel, Meyer & Matz, 2013), *Acropora tenuis* (Matz lab website) transcriptome databases and the Cnidarian Database of Centre Scientifique de Monaco (http://data.centrescientifique.mc/CSMdata-home.html). The sequences were aligned using ClustalW and degenerate primers were designed based on conserved regions. Gradient rtPCR was applied for each pair of degenerate primers using Ready Mix RedTaq reaction mix (SIGMA) or with DreamTaq Green DNA Polymerase (Thermo Scientific). Each 50 µl reaction contained 25 µl polymerase, 4 µl of each of the forward and reverse primers, 1 µl of cDNA and 16 µl ddH2O (nuclease free water). PCR temperature profiles were as manufacturer’s instructions. PCR products with the most stringent temperature that yielded a band of the suitable size upon 1% agarose gel were sent for sequencing in Hylabs or Macrogene. Resulting sequences were assembled and sequence identity was confirmed using BLAST search through the NCBI server on the GenBank database. Sequences generated in this study were deposited in GenBank under accession numbers KT957160– KT957173. These partial sequences then served as a template for specific primers design for real-time PCR primers (see Table S1), using Primer Quest design tool.

### Real-Time Polymerase chain reaction

Complementary DNAs were synthesized from 1 µg of total RNA with 1 µl Solaris RNA spike (Thermo-Scientific) using the RevertAid First Strand cDNA Synthesis kit (Thermo), according to the manufacturer’s instructions. Assuming equal RNA loading, the Solaris spike controls are designed to act as a synthetic exogenous control to identify the presence of reaction inhibition and thereby circumvents the need for a housekeeping gene (Mayfield, Hirst & Gates, 2009; Mayfield et al., 2012; Putnam et al., 2013). Spike-inoculated cDNAs were diluted 1:10 and 4 µl were used for technical triplicates of 10 µl qRT-PCR reactions with 0.5 µl mix of forward and reverse primers, 5 µl of GoTaq qPCR Master Mix (Promega) and 0.5 µl of RNAse free water, for 45 cycles. A melt curve analysis was performed for each pair of primers, to test for nonspecific amplification products by incubating the reactions for 10 s at 0.5 °C increments between 60 °C and 90 °C. Primer efficiencies were determined using a standard curve analysis with a 4-fold dilution series and according to the formula: % Efficiency =(*E* − 1) × 100% (*E* is calculated from the slope of the standard curve: *E* = 10 − 1∕slope).

The comparative ΔΔCTs method was applied, and fold changes were calculated using the 2^−ΔΔCt^ formula to estimate the relative amounts of transcripts in each sample (Livak & Schmittgen, 2001). Ct refers to the cycle at which the fluorescence signal crosses the threshold and by using the solaris spike control (Mayfield, Hirst & Gates, 2009; Mayfield et al., 2012; Putnam et al., 2013) we normalized the expression to RNA loading. The MIQE guidelines were taken into account in designing real time profiles and analyzing their results (Bustin et al., 2009).

### Statistical analysis

Results from *Fv*∕*Fm* data and gene expression were tested for normality and equal variances. In order to distinguish significant results we used the One-way ANOVA analysis followed by post hoc LSD/Bonferroni multiple-comparisons test (*p* < 0.05). All statistical analyses were performed using SPSS software (Version 20.0. Armonk, NY, IBM Corp).

### Protein oxidation assay

Protein oxidation was determined in extracts of corals fragments by measuring the degree of protein carbonylation present using Oxyblot protein oxidation kit (Millipore, Billerica, MA, USA) (see Supplemental Information 1).

## Results

### PAM fluorometer

**Figure 2:**
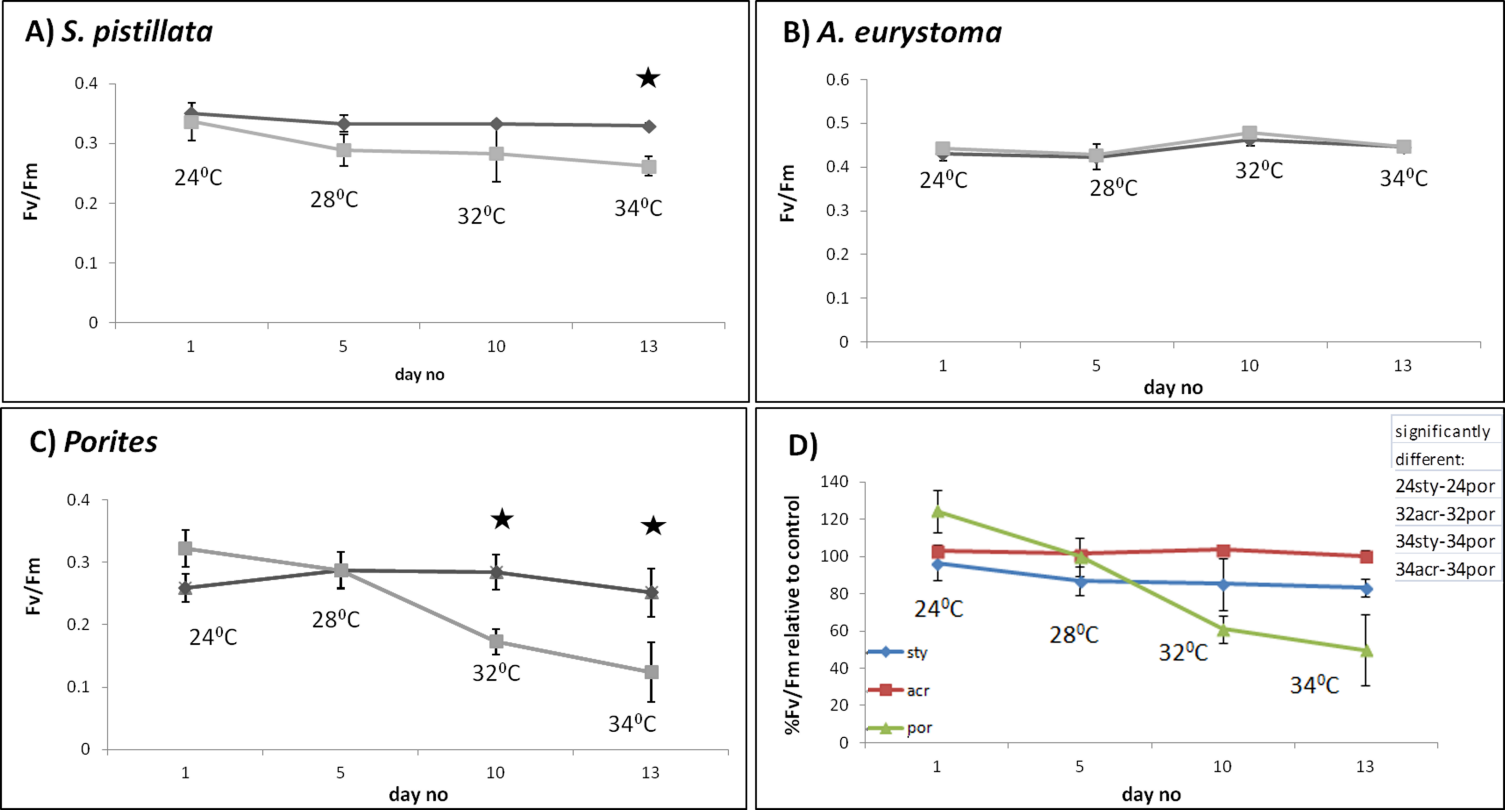
Maximum quantum yield of coral fragment symbionts throughout the experiment. Maximum quantum yield (*Fv*∕*Fm*) values for heat-stressed (light grey) and control (dark grey) coral fragments; (A) *S. pistillata*, (B) *A. eurystoma* and (C) *Porites*. Asterisk represents a significant difference between control and treatment (*p* < 0.05). (D) Percentage of *Fv*∕*Fm* relative to control for the three studied coral species, as indicated in the legend. The table in the upper right hand corner represents the significantly different treatments (using post hoc LSD multiple-comparisons) (por; Porites sp., sty; *S. pistillata*, acr; *A. eurystoma* 24, 28, 32 and 34; fragments sampled at the time points corresponding to 28 °C, 32 °C, 34 °C).

**Figure 3:**
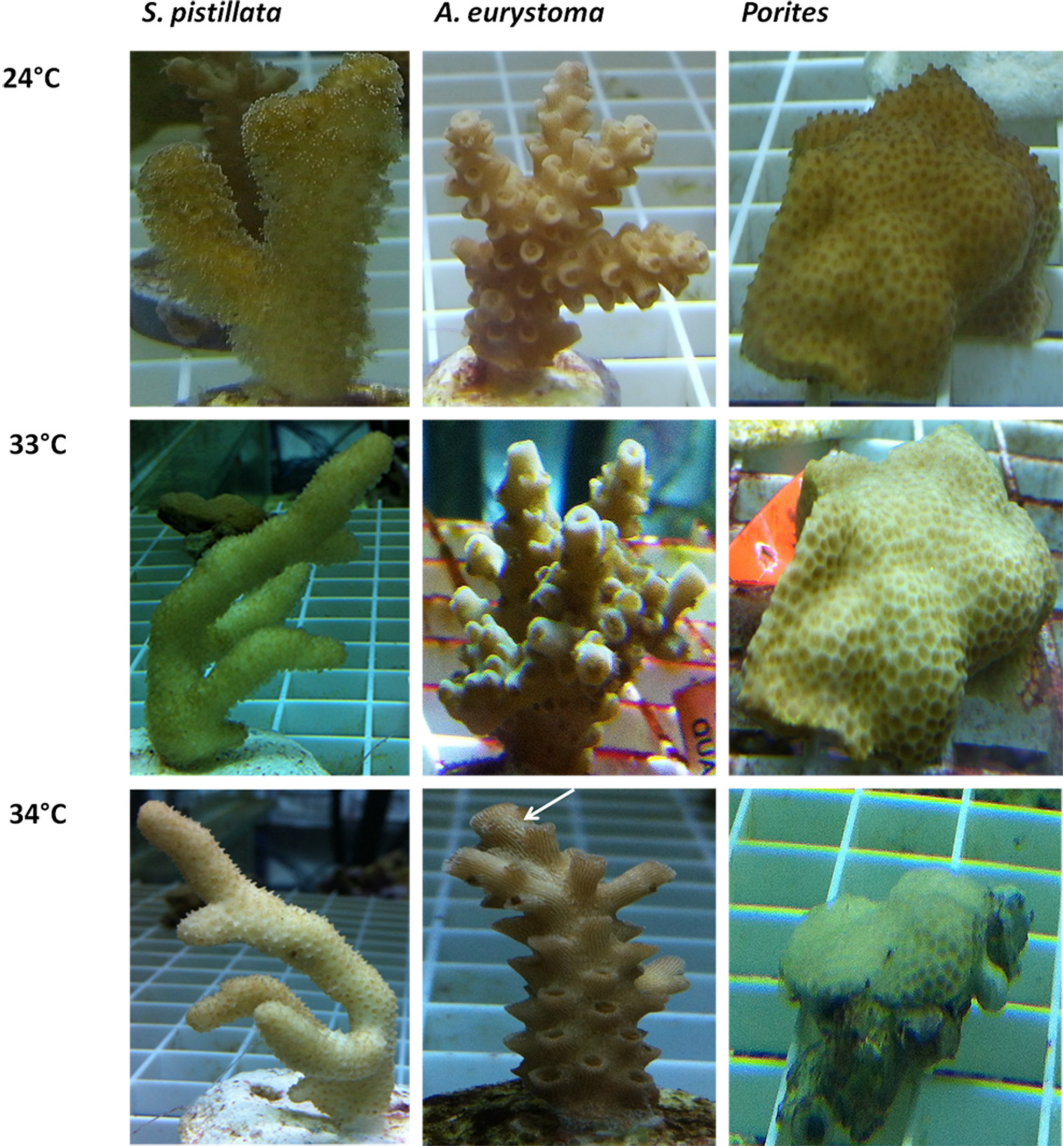
Visual appearance of coral fragments at ambient 240°C and following thermal stress of 33 °C and 34 °C. *Acropora eurystoma t*_*s*_ tissue sloughing is indicated in the figure with a white arrow.

*S. pistillata* heat stress fragments showed a decreased maximum quantum yield in comparison to control fragments only as temperature reached 34 °C (One-way ANOVA, *p* < 0.05) (Fig. 2A). The color intensity of the heat-stressed coral fragments visually appeared to fade from day 11 and bleaching was greatest at 34 °C (Fig. 3). Throughout the course of the experiment, the heat shocked *A. eurystoma*’s symbionts’ maximum quantum yield did not differ from that of the control fragments (One-way ANOVA, *p* > 0.05) (Fig. 2B). However, several of *A. eurystoma* fragments’ tissue began to peel off the skeleton by day 12 at the high temperatures. *Porites sp.* fragments exhibited a different maximum quantum yield pattern; under heat stress *Fv*∕*Fm* values deteriorated gradually from day 1 (One-way ANOVA, *p* < 0.05) (Fig. 2C). Correspondingly the color intensity became paler as time went on (Fig. 3). Figure 2D summarizes these results and presents the three coral species *Fv*∕*Fm* values relative to their respective controls. Maximum quantum yield of the coral symbionts indicates that *A. eurystoma*’s symbiont*s* are the most resilient, followed by *S. pistillata*, while the most sensitive appeared to be those of the *Porites*.

### Gene expression

**Figure 4:**
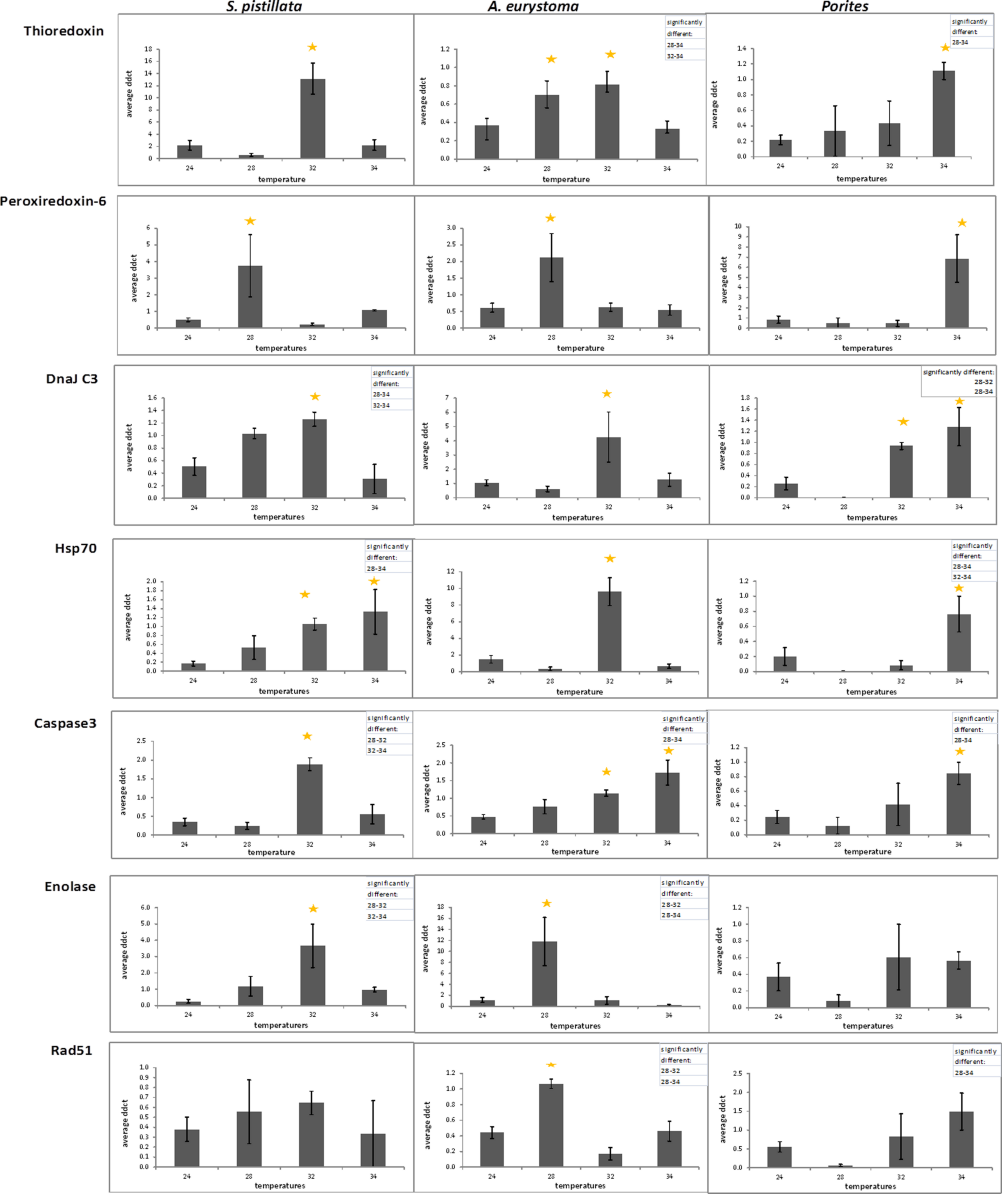
Gene expression following treatment of 28 °C, 32 °C, 34 °C and in 24 °C control treatment. Gene expression (represented as average ΔΔCT) of thioredoxin, peroxiredoxin-6, Dnaj C3, Hsp70, caspase 3, enolase and Rad51 in *S. pistillata* , *A. eurystoma* and *Porites* following treatment of 28 °C, 32 °C, 34 °C and in 24 °C control treatment. Results were subjected to One-way ANOVA followed by post-hoc LSD/bonferroni multiple-comparisons test (*p* < 0.05). Treatments significantly different from control were considered as up-regulated and indicate with an asterisk. The table in the right hand upper corner contains additional significantly different treatments.

With the purpose of evaluating gene expression, Real-Time PCR was used to quantify seven genes of interest (GOI) in the three coral species. Utilized PCR primers (Table S1) were based upon partial sequences achieved using degenerate primers. No correlation was found in all control ΔΔCTs (from fragments of two aquaria and three sampling points) between aquariums and also between sampling times in One-way ANOVA (*p* > 0.05). Since control ΔΔCTs were not different from one another, the replicates were considered to be independent and an arithmetic mean was calculated for all control values. Average values of comparative ΔΔCT are presented in Fig. 4 for heat-stress treatments showing significant gene expression differences in comparison to the average of control samples and considered to be up-regulated values (One-way ANOVA followed by post hoc multiple comparisons analyses, *p* < 0.05).

In general, GOI expression profiles were similar between *S. pistillata* and *A. eurystoma*, while for most of the cases, *Porites* exhibited a different gene expression response (Fig. 4 and Table 1). *Porites* thioredoxin, peroxiredoxin-6, hsp70 and caspase 3 were up-regulated only when the heat-stress was severe, i.e., at 34 °C. However, at 32 °C one gene, DNAJ C3 was found to be up-regulated in *Porites*. Therefore DNAJ C3 up-regulation at 32 °C represented the only common feature between the three coral species and the three temperature treatments. Enolase and Rad51 levels in *Porites* were not significantly different from the control throughout the experiment while they did differ in the other species. The redox regulation thioredoxin was up-regulated in *S. pistillata* and in *A. eurystoma* as temperature reached 32 °C, and in *A. eurystoma* the levels were elevated also at 34 °C. The additional redox regulation gene studied, peroxiredoxin-6, was up-regulated in both branched corals only at the beginning of the heat-stress at 28 °C and returned to basal level at higher temperature stresses (no different from the control, One-way ANOVA, *p* > 0.05). The molecular chaperone DNAJ present comparable results with regard to *S. pistillata* and *A. eurystoma*, elevated expression at 32 °C, though the pattern is more gradual in *S. pistillata*. Heat shock protein 70 was up-regulated in *S. pistillata* at 32 °C and 34 °C, but in *A. eurystoma* was only up-regulated at 32 °C. The apoptosis-executioner agent Caspase 3 was elevated at 32 °C in *S. pistillata*, and decrease to its basal level at 34 °C. In *A. eurystoma* Caspase 3 remained elevated as well at 34 °C. Enolase, which acts in energy metabolism in the cell, was up-regulated in the branched corals *A. eurystoma* at 28 °C and in *S. pistillata* only at 32 °C and was not up-regulated in *Porites*. The DNA repair representative, Rad51, was significantly up-regulated only at 28 °C in *A. eurystoma* (One-way ANOVA followed by post hoc multiple comparisons analyses, *p* < 0.05) but not at other temperatures or in the other corals sampled at these temperatures.

**Table 1:** Up-regulated genes in *Stylophora pistillata*, *Acropora eurystoma* and *Porites* following treatment of 28 °C, 32 °C and 34 °C, based on Fig. 4.

	*S. pistillata*			*A. eurystoma*			*Porites*		
	28 °C	32 °C	34 °C	28 °C	32 °C	34 °C	28 °C	32 °C	34 °C
Thioredoxin		↑		↑	↑				↑
peroxiredoxin-6	↑			↑					↑
Dnaj C3		↑			↑			↑	↑
Hsp70		↑	↑		↑				↑
Caspase 3		↑			↑	↑			↑
Enolase		↑		↑					
Rad51				↑					

## Discussion

To ascertain if specific corals possessing different morphologies may manifest varied gene expression patterns, we studied the expression of seven key representative genes of cellular processes known to occur during heat-stress in Cnidaria: two redox regulation agents: thioredoxin and peroxiredoxin, heat shock protein 70, Dnaj which is involved in the unfolded protein response (UPR) in ER stress, energy metabolism agent enolase, DNA repair mediator rad51, and apoptosis executioner caspase 3. The three studied coral species showed a variety of cellular responses that were correlated to their morphology as well as to their taxonomic classification.

**Figure 5:**
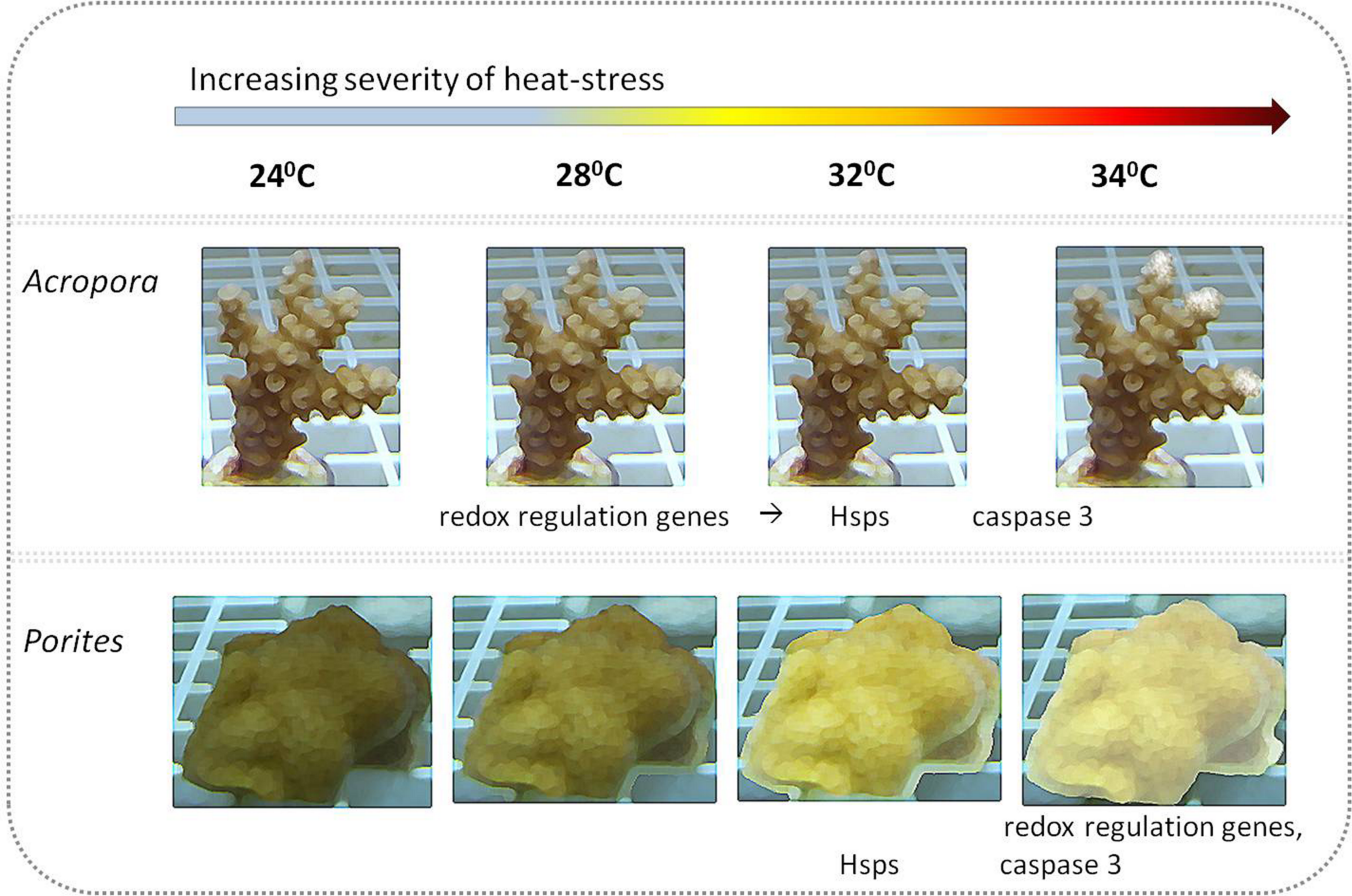
Illustration of coral visual appearance and up-regulated genes throughout the heat stress experiment in *Acropora* and *Porites* fragments. *Acropora eurystoma* fragments didn’t exhibit bleaching, but at 34 °C live tissue started to peel off the skeleton, while *Porites* fragments were paler at 28 °C and bleaching was maximized at 34 °C. Below coral figures, up-regulated genes are indicated, corresponding to the temperature they were elevated at.

A varied response to the heat stress, in terms of visual coral paling, algal maximum quantum yield and host gene expression was evident following heat stress on the coral. Overall, the two branching corals exhibited a more similar response to each other, than to the massive coral. *Fv*∕*Fm* values under elevated temperatures decreased quickly in *Porites*, while in *S. pistillata* this occurred only in the severe temperatures treatment of 34 °C while in *A. eurystoma* they remained high throughout the experiment. The visual appearance of coral bleaching corresponded with this pattern. Bearing in mind some of our gene expression results, it seems likely that *Porites* exhibited a delay in the stress response. Compared to *S. pistillata* and *A. eurystoma* genes that in most cases were up-regulated as the temperatures reached 28 °C or 32 °C, in *Porites* these genes were elevated mostly only at 34 °C, or not at all. The relative resilience of *Porites* and other massive corals to heat stress is well known in coral literature (Jokiel & Coles, 1974; Brown & Suharsono, 1990; Loya et al., 2001). Here we showed that *Porites* displayed severe bleaching under elevated temperature along with a postponed molecular gene expression response to stress. We postulate that by expelling the algal symbionts from its gastrodermal tissues, oxidation damage in the *Porites* may be reduced and thus coral animal tissue associated stress genes may be activated only at a later stage. On the other hand, *A. eurystoma*, a species that is considered to be susceptible to heat stress, did not bleach throughout our experiment and correspondingly both of the redox regulation genes were up-regulated already at the beginning of the experiment, at temperatures as low as 28 °C (see Fig. 5). Interestingly, this species began losing its tissues at the elevated temperatures, perhaps as a response to accumulations of free radicals in their tissues. *S. pistillata* may represent an intermediate version of the two, with bleaching occurring only in extreme temperatures and the redox regulation thioredoxin being up-regulated not early as in *A. eurystoma* but sooner than in *Porites*. Coral bleaching was previously suggested in the literature as a host resort for survival; expelling or degrading the compromised ROS-causing symbionts and breakdown of the symbiosis (Downs et al., 2002; Downs et al., 2009). This study demonstrates how gene expression may reflect this characteristic.

The two relatively heat-stress sensitive coral species of this study showed elevated levels of caspase 3 at 32 °C. Members of the family of caspases—cysteine-dependent aspartate specific proteases—are the core effectors of the apoptotic cascade (Nicholson & Thornberry, 1997) that cleave a variety of cellular subtracts resulting in programmed cell death (Chowdhury, Tharakan & Bhat, 2008). Caspase 3 is an executioner caspase that was studied in corals with regards to gene expression (Kvitt et al., 2011; Tchernov et al., 2011; Kaniewska et al., 2012; Shearer et al., 2012) and enzyme specific activity (Pernice et al., 2011; Hawkins et al., 2014). In the present study, *S. pistillata*, caspase-3 expression increased with elevated temperature and then decreased to basal levels at 34 °C. This result resembles results of a chronic heat-stress study previously conducted on *S. pistillata* (Kvitt et al., 2011). In that study the decrease in caspase-3 was attributed to acclimatization of the coral to the chronic heat stress together with the completion of symbiosis breakdown (Kvitt et al., 2011). In *A. eurystoma*, caspase-3 levels remained elevated at 34 °C, suggesting an inability to acclimatize. This response is also reflected in the peeling off of the live tissue at this temperature. These results rank *A. eurystoma* as the most susceptible species to heat-stress in this experiment. As opposed to caspase 3 in *S. pistillata* and *A. eurystoma*, in *Porites* caspase-3 was elevated only when the heat-stress was most severe, at 34 °C, which may explain the higher resilience observed in this coral.

The gene expression patterns of enolase, a representative of energy regulation process, and rad51, an agent of DNA repair process, differed greatly between the coral species. There are only few reports in the literature indicating the role of these genes following environmental stress in corals. These include the association of enolase with heat stress, or with macroalgal exposure (Maor-Landaw et al., 2014; Shearer et al., 2012). The association of rad51 expression with UV radiation exposure in coral larvae was also reported (Aranda et al., 2011). There are several possible explanations to these results; (a) the sampling points may have missed the maximal gene expression point (e.g., gene expression peaks at 30 °C); (b) Enolase and rad51 genes may not be suitable or prominent representatives of these stress processes in coral cells; or (c) The genes are good representative of stress response in these pathways, but the processes themselves of higher energy demands and DNA repair are not occurring in this experiment. These possible interpretations can be relevant to one of the species or common to all. A fundamental issue is whether the genes that govern these cellular processes in the different coral species are the same and only the timing varies, or alternatively, the key players in mitigating stress are different. The above-mentioned discrepancies still need to be resolved.

In contrast, the two-redox regulation agents and the two heat shock proteins studied here, were all up-regulated at some point in all the three coral species. Therefore, these may provide suitable candidates as markers of redox regulation and heat shock processes in the three corals. Thioredoxin, an enzyme that detoxifies oxidized molecules, was previously reported to be up-regulated in corals following thermal stress (DeSalvo et al., 2010a; Maor-Landaw et al., 2014), high irradiance (Starcevic et al., 2010), macroalgal exposure (Shearer et al., 2012), and elevated salinity (Edge et al., 2005). Peroxiredoxin elevation was also previously documented after heat-stress (Maor-Landaw et al., 2014), and also was related to white band disease in *Acropora* (Libro, Kaluziak & Vollmer, 2013). Hsp70 is known to be an important factor in protein folding and repair of stress-induced protein damage (Tavaria et al., 1996) and is well documented during coral stress (Brown et al., 2002; Carpenter, Patterson & Bromage, 2010; Putnam et al., 2012; Barshis et al., 2013). DNAJ, also termed hsp40, expression, was previously reported to be heat-stress related in the coral *Acropora* (DeSalvo et al., 2010b; Yuyama et al., 2012) and in *S. pistillata* (Maor-Landaw et al., 2014). The results presented here, showed that DNAJ up-regulation at the time point corresponding to 32 °C is the only common temperature related expression feature and timing of all the three corals species. This marker may thus provide an important potential biomarker for early warning detection of heat stress, as suggested by our study on scleractinian corals of Eilat. DNAJ plays an important role in the unfolded protein response (UPR) during ER stress and also serves as a co-chaperone to hsp70 (Cyr, Langer & Douglas, 1994) indicating that it may be a suitable marker for heat stress. In the Eilat *S. pistillata,* 32 °C was previously suggested to be the temperature of initial stress reaction (Maor-Landaw et al., 2014) and 34 °C as the upper thermal limit (Shaish, Abelson & Rinkevich, 2007; Kvitt et al., 2011). This may differ with ambient temperature regime of these populations as colonies of this species are known to flourish in much warmer waters (Bauman, Baird & Cavalcante, 2011). Indeed DNAJ is elevated at 32 °C in *S. pistillata* before bleaching occurs, before tissue peeling off in *A. eurystoma* and before most of the gene expression heat-stress response in *Porites*.

Protein carbolyation a common marker of protein oxidation of stress-induced damage (Murik & Kaplan, 2009) was used to estimate protein oxidation levels (Supplemental Information 1 ). The results indicated that protein oxidation differed between species. In *Porites sp* the profile of protein oxidation following 34 °C treatment did not significantly differ from the 24 °C control. In *S. pistillata* maximum protein oxidation was at 34 °C. In *A. eurystoma* protein oxidation peaked at 32 °C (see Figs. S1 and S2). This pattern is comparable with our previous results and with the hierarchy documented in the literature (Jokiel & Coles, 1974; Brown & Suharsono, 1990; Loya et al., 2001). It also provides additional explanation for the resilience of “winner” corals with massive-morphology (*Porites sp.)*, when compared to that of “looser” branching corals *S. pistillata* and especially *A. eurystoma*, that are more sensitive to heat stress.

The corals studied here representing different growth forms, S*. pistillata*, *A. eurystoma* and *Porites sp.*, demonstrated different physiological response to short-term heat stress. These responses included visual coral paling and algal maximum quantum yield, and varied host gene-expression reactions to elevated temperature.

We acknowledge the possible role of zooxanthellae in the thermal tolerance of corals (Berkelmans & Van Oppen, 2006); however, this was not the scope of our research. Most of the corals of the Gulf of Eilat host *Symbiodinium* clade A or C that are both known to be relatively sensitive to heat stress (Karako-Lampert et al., 2004; Lampert-Karako et al., 2008; Fine, Gildor & Genin, 2013). Lately, symbiont enzymatic antioxidant activity was found to be independent of thermal sensitivity (Krueger et al., 2015), so the dispute over the potential coupling of symbiont antioxidant capacity and bleaching outcome (Hawkins et al., 2015) is still ongoing.

In *Porites sp.* early-stage bleaching corresponded with a delayed response of redox regulation agents, heat shock proteins, and caspase 3. At the other end of the spectrum the literature-know relatively susceptible *A. eurystoma*, did not bleach throughout the experiment, oxidative damage was manifested in its cells leading ultimately to programmed cell death (Fig. 5). The differentially expressed gene responses of the studied branching and massive coral species can be correlated with the literature of well-documented hierarchy of susceptibilities amongst coral morphologies and genera in Eilat’s coral reef. For a more comprehensive understanding of this phenomenon further investigations should be undertaken by comparing conspecific corals with different growth patterns such as branching vs. massive *Porites* or with conspecific from environments with different natural temperature ranges. Future studies should consider looking deeply into the plasticity of a coral in expelling symbiont process as an approach to elevate heat stress resistance, which was not the main scope of this research.

## Supplemental Information

10.7717/peerj.1814/supp-1 Supplemental Information 1 Supplementary information

10.7717/peerj.1814/supp-2 Table S1 Primers and PCR conditions for qRT-PCR Primers and PCR conditions for qRT-PCR (thio; tioredoxin, pero; peroxired oxin-6, hsp; hsp70, dnaj; Dnaj C3, casp3; caspase 3, eno; enolae, rad; rad51, F; forward, R; reverse).

10.7717/peerj.1814/supp-3 Figure S1 Protein oxidation in *Porites sp.*, *Stylophora pistillata* and *Acropora eurystoma* following heat-stress of 28, 32 and 34 °C and control of 24 °C. Protein oxidation in *Porites sp.*, *Stylophora pistillata* and *Acropora eurystoma* following heat-stress of 28, 32 and 34 °C and control of 24 °C. Detection of proteins containing carbonyl groups (indicative of protein oxidation) was performed by Oxyblot kit and a protein-blot assay. (por; Porites sp., sty; *S. pistillata*, acr; *A. eurystoma*, 24, 28, 32 and 34; fragments sampled at the time points corresponding to 28 °C, 32 °C, 34 °C).

10.7717/peerj.1814/supp-4 Figure S2 Densitometry of protein carbonylation assay. Densitometry of protein carbonylation assay. ImageJ software was used to quantify protein oxidation profiles of *Porites sp.*, *Stylophora pistillata* and *Acropora eurystoma* following heat-stress of 28, 32 and 34 °C and control of 24 °C. All densitometry results were normalized to densitometry of total protein output of commasie brilliant blue staining. (por; Porites sp., sty; *S. pistillata*, acr; *A. eurystoma*, 24, 28, 32 and 34; fragments sampled at the time points corresponding to 28 °C, 32 °C, 34 °C).

## Additional Information and Declarations

### Field Study Permissions

The following information was supplied relating to field study approvals (i.e., approving body and any reference numbers): The Israeli Nature and National Parks Protection Authority approved the collection of corals in this study, permit No. 2013/40159.

### DNA Deposition

The following information was supplied regarding the deposition of DNA sequences: Sequences generated in this study were deposited in GenBank NCBI under accession numbers KT957160– KT957173.

### Data Availability

The following information was supplied regarding data availability: The research in this article did not generate any raw data.
