# Supplementary material for: Gene expression profiles during short-term heat stress; branching vs. massive Scleractinian corals of the Red Sea
Source: PeerJ. 2016 Mar 28;4:e1814. doi: 10.7717/peerj.1814 (PMC4824894; doi:10.7717/peerj.1814)
Supplement: Supplemental Information 1 [file peerj-04-1814-s001.docx]

**Gene expression profiles during short-term heat stress; branching vs. massive Scleractinian corals of the Red Sea**

**SI**

Keren Maor-Landaw^1^ and Oren Levy^1*^

^1^ The Mina and Everard Goodman Faculty of Life Sciences, Bar Ilan University, Ramat Gan, Israel.

*Corresponding author – Oren Levy;

email: [oren.levy@biu.ac.il](mailto:oren.levy@biu.ac.il)

Telephone: +97235318030

Fax: +97237384181

**Materials and methods**

**Protein extraction**

Protein was extracted from remaining coral fragments of *S. pistillata* following treatments of 28, 32 and 34^0^C, *A. eurystoma* following 32 and 34^0^C and *Porites sp.* following 34^0^C. Protein was extracted as well from the corresponding control fragments which were maintained at 24^0^C. On ice, the tissue was removed of the skeleton by a fine toothbrush into chilled 1-2 ml of phosphate buffered saline (PBSX1). The lysates were homogenized and centrifuged at 4000 rpm, 4^0^C for 10 minutes. The supernatant with host protein was collected. Total protein concentration of each sample was measured using the Bradford protein assay and BSA protein standard (both from BioRad, CA, USA) according to the manufacturer’s instructions.

**Protein Oxidation Assay**

Protein oxidation was determined in extracts of corals fragments by measuring the degree of protein carbonylation present using Oxyblot protein oxidation kit, as recommended by the manufacturer (Millipore, Billerica, MA) (Tunc-Ozdemir et al., 2009; Miller et al., 2007, Murik and Kaplan, 2009). Ten micrograms of each protein extract were reacted with 2,4-dinitrophenylhydrazone (DNPH) to label carbonylated amino acid residues on oxidized proteins. DNPH-labeled protein extracts were fractionated by 12.5% SDS-PAGE and further transferred to a PVDF membrane. Following blocking with BSA 1%, the membrane was incubated with Rabbit Anti-DNP antibody (1:150 dilution) for 4 hr. A Cross-reacting protein bands to rabbit anti-DNP antibody were visualized using Goat Anti-Rabbit IgG (HRP-conjugated) (1:300 dilution) and chemiluminescence detection. Blots were analyzed using densitometry-analysis software (ImageJ - <http://rsb.info.nih.gov/ij/>) and normalized to total protein output of commasie brilliant blue staining.

**Results**

Protein oxidation was visible and varied between coral species and treatments (Figures S1 and S2). By comparing each treatment to the control, within the coral species, we can indicate a least effect on *Porites sp.* at 34^0^C. *S. pistillata* protein oxidation was maximized at 34^0^C, while *A. eurystoma* already as temperatures reached 32^0^C showed the largest effect.

**References**

Miller, G., Suzuki, N., Rizhsky, L., Hegie, A., Koussevitzky, S., and Mittler, R. (2007). Double mutants deficient in cytosolic and thylakoid ascorbate peroxidase reveal a complex mode of interaction between reactive oxygen species, plant development, and response to abiotic stresses. *Plant Physiol.* 144, 1777–1785. doi:10.1104/pp.107.101436.

Murik, O., and Kaplan, A. (2009). Paradoxically, prior acquisition of antioxidant activity enhances oxidative stress-induced cell death. *Environ. Microbiol.* 11, 2301–9. doi:10.1111/j.1462-2920.2009.01957.x.

Tunc-Ozdemir, M., Miller, G., Song, L., Kim, J., Sodek, A., Koussevitzky, S., Misra, A. N., Mittler, R., and Shintani, D. (2009). Thiamin confers enhanced tolerance to oxidative stress in Arabidopsis. *Plant Physiol.* 151, 421–432. doi:10.1104/pp.109.140046.
