# Supplementary material for: Gene expression profiles during short-term heat stress; branching vs. massive Scleractinian corals of the Red Sea
Source: PeerJ. 2016 Mar 28;4:e1814. doi: 10.7717/peerj.1814 (PMC4824894; doi:10.7717/peerj.1814)
Supplement: Table S1 — Primers and PCR conditions for qRT-PCR (thio; tioredoxin, pero; peroxired oxin-6, hsp; hsp70, dnaj; Dnaj C3, casp3; caspase 3, eno; enolae, rad; rad51, F; forward, R; reverse). [file peerj-04-1814-s002.docx]

Table S1 – Primers and PCR conditions for qRT-PCR (thio; tioredoxin, pero; peroxiredoxin-6, hsp; hsp70, dnaj; Dnaj C3, casp3; caspase 3, eno; enolae, rad; rad51, F; forward, R; reverse).
