# Supplementary figures and images for: Gene expression profiles during short-term heat stress; branching vs. massive Scleractinian corals of the Red Sea
Source: PeerJ. 2016 Mar 28;4:e1814. doi: 10.7717/peerj.1814 (PMC4824894; doi:10.7717/peerj.1814)

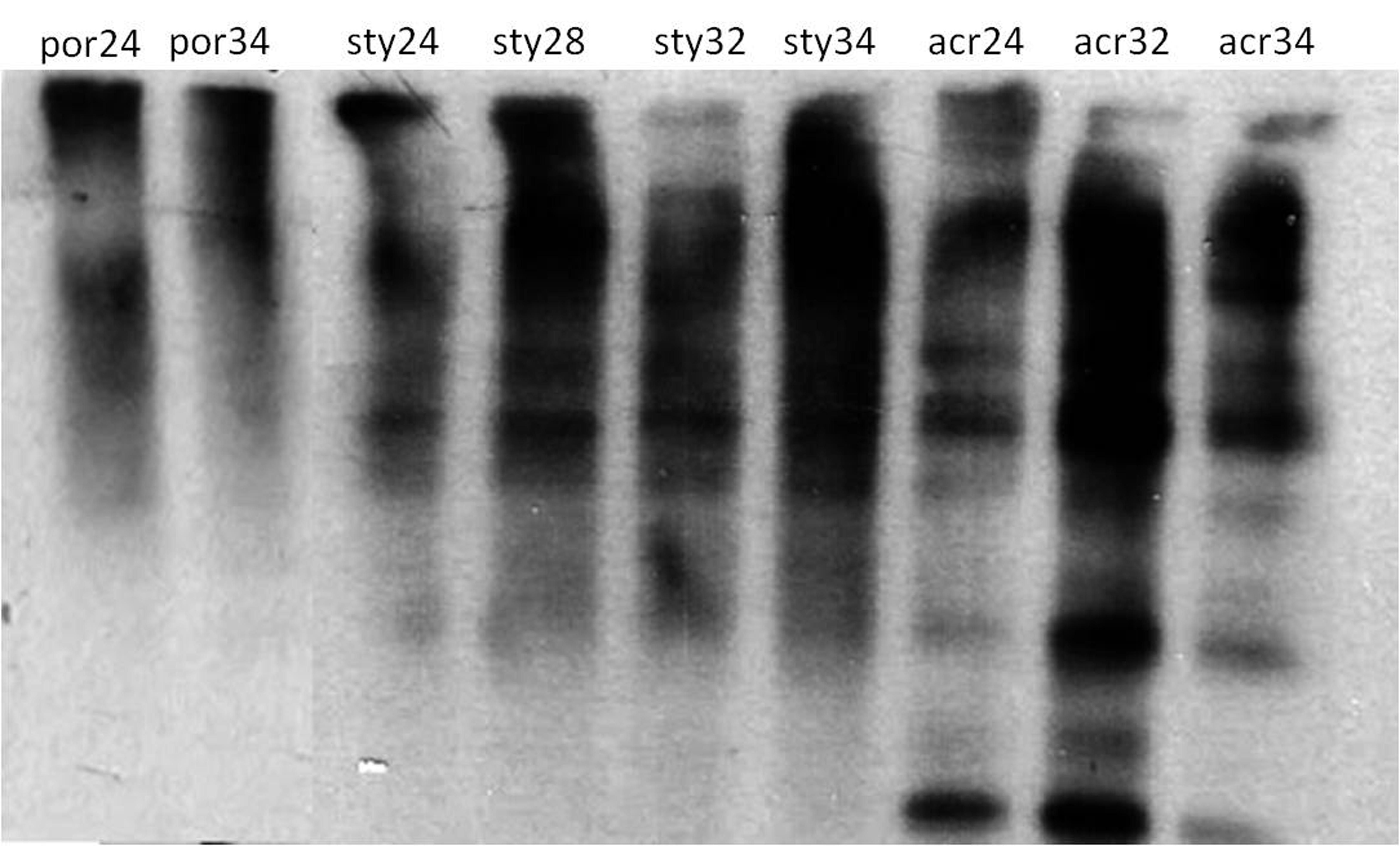

Supplement: Figure S1 — Protein oxidation in Porites sp., Stylophora pistillata and Acropora eurystoma following heat-stress of 28, 32 and 34 °C and control of 24 °C. Detection of proteins containing carbonyl groups (indicative of protein oxidation) was performed by Oxyblot kit and a protein-blot assay. (por; Porites sp., sty; S. pistillata, acr; A. eurystoma, 24, 28, 32 and 34; fragments sampled at the time points corresponding to 28 °C, 32 °C, 34 °C). [file peerj-04-1814-s003.png]

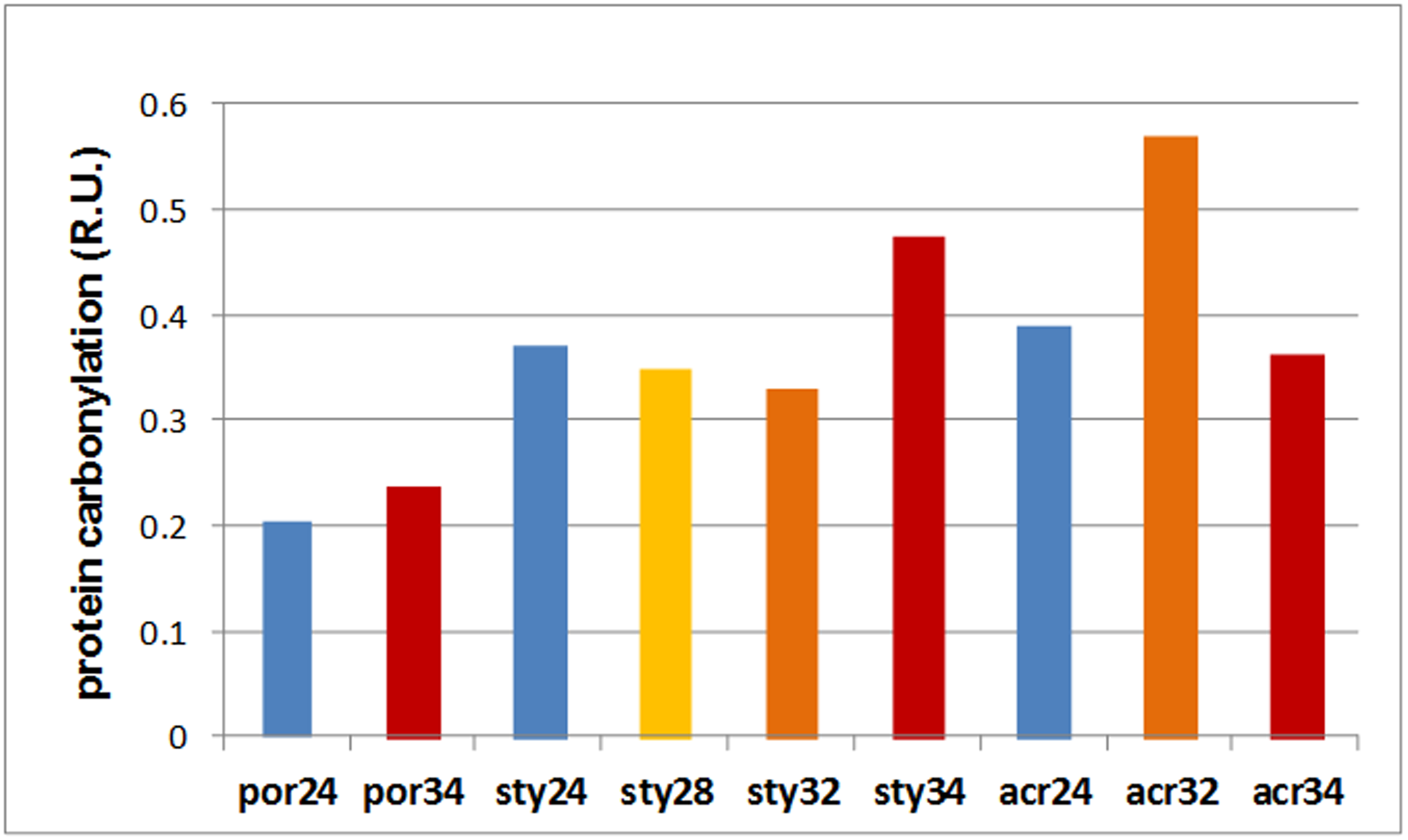

Supplement: Figure S2 — Densitometry of protein carbonylation assay. ImageJ software was used to quantify protein oxidation profiles of Porites sp., Stylophora pistillata and Acropora eurystoma following heat-stress of 28, 32 and 34 °C and control of 24 °C. All densitometry results were normalized to densitometry of total protein output of commasie brilliant blue staining. (por; Porites sp., sty; S. pistillata, acr; A. eurystoma, 24, 28, 32 and 34; fragments sampled at the time points corresponding to 28 °C, 32 °C, 34 °C). [file peerj-04-1814-s004.png]
